# The Role of Bacterial and Fungal Human Respiratory Microbiota in COVID-19 Patients

**DOI:** 10.1155/2021/6670798

**Published:** 2021-02-23

**Authors:** Saber Soltani, Armin Zakeri, Milad Zandi, Mina Mobini Kesheh, Alireza Tabibzadeh, Mahsa Dastranj, Samireh Faramarzi, Mojtaba Didehdar, Hossein Hafezi, Parastoo Hosseini, Abbas Farahani

**Affiliations:** ^1^Department of Virology, School of Public Health, Tehran University of Medical Sciences, Tehran, Iran; ^2^Research Center for Clinical Virology, Tehran University of Medical Sciences, Tehran, Iran; ^3^Department of Hematology, School of Medicine, Tarbiat Modares University, Tehran, Iran; ^4^Department of Virology, School of Medicine, Iran University of Medical Science, Tehran, Iran; ^5^Infectious and Tropical Diseases Research Center, Hormozgan Health Institute, Hormozgan University of Medical Sciences, Bandar Abbas, Iran; ^6^Razi Vaccine and Serum Research Institute, Agricultural Research, Education and Extension Organization (AREEO), Karaj, Iran; ^7^Department of Medical Parasitology and Mycology, School of Medicine, Arak University of Medical Sciences, Arak, Iran; ^8^Department of Dermatology, School of Medicine, Hormozgan University of Medical Sciences, Bandar Abbas, Iran

## Abstract

Recently, severe acute respiratory syndrome coronavirus 2 (SARS-CoV-2), the etiologic agent of coronavirus disease 2019 (COVID-19), has led to a worldwide pandemic with millions of infected patients. Alteration in humans' microbiota was also reported in COVID-19 patients. The alteration in human microbiota may contribute to bacterial or viral infections and affect the immune system. Moreover, human's microbiota can be altered due to SARS-CoV-2 infection, and these microbiota changes can indicate the progression of COVID-19. While current studies focus on the gut microbiota, it seems necessary to pay attention to the lung microbiota in COVID-19. This study is aimed at reviewing respiratory microbiota dysbiosis among COVID-19 patients to encourage further studies on the field for assessment of SARS-CoV-2 and respiratory microbiota interaction.

## 1. Introduction

The severe acute respiratory syndrome coronavirus 2 (SARS-CoV-2), as a novel coronavirus, is spreading from China and is known to be the etiologic agent for coronavirus disease 2019 (COVID-19) [1–3]. The SARS-CoV-2 belongs to *betacoronavirus* genera and phylogenetically is relevant to SARS-CoV [4]. The SARS-CoV-2 can exploit the angiotensin-converting enzyme 2 (ACE2) for priming Spike (S) protein [5, 6]. The ACE2 is expressed in the esophagus, lungs, liver, and intestinal epithelium [7, 8]. SARS-CoV-2 infection can be asymptomatic or can cause a wide spectrum of signs and symptoms: fever, dry cough, shortness of breath, pneumonia, pulmonary edema, acute respiratory distress syndrome (ARDS), multiple organ failure, and death [9]. In some patients, common symptoms include headache, nausea, and vomiting, and diarrhea is also reported [10].

At the infancy age, various bacteria, fungi, and viruses colonize in the skin, oral cavity, and gut. These microorganisms are known as the human microbiota [11–13]. The predominant human oral microbiota is summarized in Table 1. The microbiome plays an essential role in human physiology, and it is considered an important factor for the maintenance of human health [14]. Typically, these microbes are commensal or mutualists, and they help to digest food and even provide immunity [15]. As mentioned before, microbial communities are found throughout the human body; there are specialized bacterial communities in certain regions of the respiratory system that are believed to play a significant role in preserving human health [16].

The essential factor for upper respiratory tract (URT), lower respiratory tract (LRT), or disseminated respiratory infections is colonization in the URT [17]. Variations in lung microbiota could potentially improve immune response against viral and secondary bacterial infection [18]. Recent studies have shown the lung's microbiota contributed to the immunologic homeostasis and potentially altered viral infection susceptibility [19]. The ARDS is a severe complication of COVID-19 [19]. Studies showed that the lung microbiota of the patients with ARDS is different from those without ARDS [20]. This fact could be an essential issue in COVID-19 progress.

## 2. Respiratory Bacterial and Fungal Microbiota

The oral cavity can be considered as the main route of entry for different pathogens. Various microorganisms, including bacteria, fungi, viruses, archaea, are colonized in the oral cavity and termed oral microbiota [21, 22]. Temperature (37°C), saliva pH (6.5-7), and humidity of the oral cavity make an appropriate environment for microorganism survival and maintenance [23, 24]. Furthermore, oxygen availability and consuming different food with acidic or alkaline pH can influence oral organism's growth pattern. Bacterial and fungal are primary microbiota communities of the oral cavity. Six strains of bacteria include *Firmicutes*, *Bacteroidetes*, *Proteobacteria*, *Actinobacteria*, *Spirochaetes*, and *Fusobacteria*, make up 94% of the oral bacteria community, while the major fungal population includes *Candida* species followed by *Cladosporium* spp., *Aureobasidium* spp., and *Saccharomycetales* in healthy cases [25, 26].

Commensal, symbiotic, and potentially pathogenic bacteria and fungi are in equilibrium. Poor oral hygiene such as periodontitis and dental caries, also pathogens like the Epstein–Barr virus (EBV), cytomegalovirus (CMV), smoking, drinking, and antibiotic consumption can compromise this ecological balance [27–30]. Microbial either plankton or biofilm habitats are found in the oral cavity; for instance, lingual microbiota contains stable multilayers of biofilms. Microbiota in the saliva is considered plankton and cannot be due to saliva being fluid and swallowed continuously [31]. On the other hand, saliva contains proteins such as mucins, agglutinin, and proline-rich proteins that help microbial adhesions to hard tissue like teeth [32]. Using high-precision sequencing methods introduces the human oral microbiome as a part of the Human Microbiome Project. This particular field is divided into two parts: (i) core: shared in all individuals. Among all the microbiota in the body, four-strains were found more frequently than others: *Actinobacteria*, *Firmicutes*, *Proteobacteria*, and *Bacteroidetes* [33]. (ii) Variable is dependent on lifestyle and environmental determinants and is variable between individuals [23].

Moreover, the diversity of microbiota changes is highly influenced by age. Alteration in the microbiota begins at birth, for instance, the delivery route of the baby. This change in the types of microbiota in infants is less than in adults (due to the absence of hard dental tissues, only feeding by breast milk/formula and so on) and is observed until later ages [32, 34]. Microbiota maturation by biological or passive changes due to vaccines, antibiotics, viral infection, teeth decay/filling, and different disease alerts gradually [28, 35–37]. Common oral diseases like dental caries, gingivitis, and oral mucosal disease are caused by endogenous bacteria [38]. Pathogenic viruses act as exogenous factors to make dysbiosis. Ling et al. indicated that hepatitis B virus (HBV) infection elevated *Fusobacterium*, *Filifactor*, *Eubacterium*, *Parvimonas*, and *Treponema* in the oral cavity leading to the unpleasant smell of mouth [39]. Also, dysbiosis of bacterial colonization in the respiratory tract and oral cavity was induced by the H1N1 influenza virus, leading to secondary bacterial infection [18].

### 2.1. The Microbiota of the Oral Cavity

The oral cavity consists of soft tissues (including lips, soft palate, tonsil, and tongue), saliva, and hard tissue, e.g., teeth. It harbors a high diversity of microbial organisms, and each tissue contains its specialized microbial community. Mucosal surfaces have monolayers of microorganisms compared with the tongue that has thick biofilms [40].

### 2.2. The Microbiota of the Oropharynx

The oropharynx is located in soft palate and upper of the epiglottis. Microbiota of the oropharynx in healthy adults is similar to other mucosal surfaces in the oral cavity and colonized by members of *Firmicutes*, *Proteobacteria*, and *Bacteroidetes* (including *Streptococcus*, *Neisseria*, *Haemophilus*, and *Lachnospira* spp.) [18, 41–43].

### 2.3. The Microbiota of the Laryngopharynx

The salivary microbiota after the oropharynx drain into the laryngopharynx. Indeed, it connects the upper aerodigestive tract to the digestive tract. The *Firmicutes*, *Fusobacteria*, *Proteobacteria*, *Actinobacteria*, and *Bacteroidetes* were reported as the primary bacterial population in this site [44].

## 3. Physiologic Features of Respiratory Microbiota

Over the past two decades, many studies have examined the impact of oral microbiota on disease or human health. The oral tissues use some mechanisms and molecules to balance the oral flora and potential pathogens. Microbial communities are tissue-specific, which can tolerate the dominant physicochemical environment. The microbiota adhere to the epithelial surfaces' mucosal membrane and can resist the saliva flow [38]. However, the saliva flow plays a role in host defense and contains antimicrobial peptides, lysozyme, lactoferrin, defensins, and lactoperoxidase to prevent microbial overgrowth [45–48]. Immunomodulation of commensals is another mechanism to maintain the oral host-microbe balance. The epithelial cells are natural physical barriers against pathogens, and they secrete antimicrobial mediators like IL-6, IL-8, TNF-*α*, IL-1*β*/*α*, defensins, and cathelicidin LL-37 [49]. The formation of pores on the bacterial cytoplasmic membrane is considered as a significant role of defensins and LL-37. *α*/*β*–defensins are found in all oral tissues, saliva, and gingival crevicular fluid.

Defensins as antimicrobial functions can induce chemotactic ability to recruit monocytes, macrophages, and even T cells [50, 51]. Among immune cells that are involved in healthy oral immunity responses, neutrophils serve the main role. In healthy junctional epithelial tissue, LL-37 and defensins attract neutrophils. This attraction leads to migrated neutrophils that lie in the gingival margin to make a barrier against dental plaque germs [52]. Commensals also control neutrophil migration in gingival tissues through modulating intracellular adhesion molecule 1 (ICAM-1) and E-selectin expression [53]. Neutrophils can generate nitric oxide and nitrogen intermediates with protective effects against bacteria [54]. IL-17-mediated immunity contributes to mucosal fungal surveillance, especially *Candida* spp. In parallel, IL-17 enhances the epithelium integrity via regulation of claudin, promotes the antimicrobial peptides expressed by epithelial cells, and elicits the secretion of neutrophil chemotaxis [49, 55]. The point to consider is that the commensal bacteria inhibit IL-17 family members' overexpression in a negative feedback manner to keep the oral homeostasis [56].

The other mechanism is bacteriophages that regulate the oral ecosystem as biocontrollers. Endodontic infection caused by *Enterococcus faecalis* could be healed through bacteriophages [57, 58]. Lytic bacteriophages can lyse bacteria and alleviate the bacterial pathogen numbers. The released substances from lysed bacteria also activate the immunity responses. These findings led to defining a concept called “immunophage synergy” [59]. Besides, bacteriophages have a direct impact on host immunity, either adaptive or innate immunity. Macrophages and dendritic cells can take up the bacteriophages as a virus or with their hosts and, consequently, induce cytokine responses. They also act as opsonin molecules to cover bacterial cytoplasmic membrane to stimulate phagocytosis. Commensal bacteriophages induce specific anti-phage antibodies. Specific anti-T4 phage IgG against viral gp24 and gp23 proteins was found in sera of healthy subjects [60, 61].

The presence of multiple species can give balance to populations of microorganisms in the body, e.g., *Pichia* in the oral cavity has an antagonistic relation with *Aspergillus*, *Fusarium*, and particularly *Candida*. Sometimes competition for nutrient uptake can limit germination and adhesion. A decrease in *Pichia* amount accompanies by increase in the growth of opportunistic fungi [62]. Bacteria use quorum sensing to communicate with other bacteria. Antagonistic interactions occur between *Porphyromonas gingivalis* (*Pg*), *a periodontal pathogen and normal flora Streptococcus Gordonii* (*S. Gordonii*), *Streptococcus intermedius*, and *Streptococcus mitis*. Arginine deiminase, encoded by the ArcA gene in these commensals, decreases expression of FimA that is a virulence factor in *Pg*. Hydrogen peroxidase produced by these streptococci can limit *P. gingivalis* growth in oral cavity [63]. Due to the lack of catalases in *S. Gordonii*, *Actinomyces naeslundi* breaks down the H_2_O_2_ generated by *S. Gordonii*. A symbiotic relationship is present between these two bacteria while competes with other possible pathogens [64, 65]. A competition between commensals and *Streptococcus mutans* (*S. mutans*) *was* suggested. Commensals overcome *S. mutans* by alkali components like urea to nearly provide a neutral environment [66]. Further, serine protease challisin derived from S. *Gordonii* interferes and degrades *S. mutans* bacteriocin production [67].

## 4. Pathogenesis of Respiratory Microbiota

Periodontitis, defined as destructive gum infection with tooth attachment loss and severe inflammation, is mainly caused by *Porphyromonas gingivalis* (*P. gingivalis*). Pg's adherence is mediated by a virulence gene known as *FimA* [68]. *P. gingivalis* also harbors *dpp* genes, which code dipeptidyl peptidases (DPP) [69, 70]. Interestingly, *dpp* genes present in subgingival crevice colonized bacteria, but not in mucosal surfaces and tongue isolated bacteria [71]. The high DPP4 activity was observed in the saliva of patients with chronic periodontitis [72]. DPP4 can degrade incretin hormones released in response to fat and glucose ingestion by increasing insulin secretion. However, the effect of insertion is not seen in people who have type 2 diabetes [71, 73, 74]. *P. gingivalis* through *α*5*β*1-integrin expressed on the epithelial cells, crosses the epithelial barrier, and enters the bloodstream [75]. LPS from *P. gingivalis* activates the TLR-4 signaling and triggers the secretion of IL-1*β* and IL-6 [76, 77]. TLR-4 signaling activated by *Pg* is also reported to be associated with human pancreatic tumors [78]. Moreover, anti-*P. gingivalis* antibodies in mouse model of periodontitis were able to prevent mice developing metabolic diseases [69]. Viral infections such as Herpes simplex virus-1, cytomegalovirus, and EBV virus can impair or suppress the immune system and induce aggressive periodontitis. A cooperative complex of *Pg*, *S. aureus*, and Herpes simplex-1 accelerates aggressive periodontitis [79]. Kaposi's sarcoma-associated herpesvirus (KSHV) is known as the most common AIDS-associated tumor [80]. The lipoteichoic acid (LTA) of *S. aureus* and lipopolysaccharide (LPS) of *Pg* can facilitate entry of KSHV through upregulation of heparan sulfate and heparan sulfate proteoglycans (viral receptors) and induce reactive oxygen species production (ROS). The LTA and LPS established viral latency by increasing viral latency-associated nuclear antigen (LANA) expression [81]. These findings suggested the role of *Pg* as a periodontal microbiota on the immune system and systemic diseases.

On the other hand, other periodontal pathogens, *Fusobacterium*, *Prevotella*, and *Alloprevotella* were enriched in HPV-negative in nonsmokers patients with oral cavity squamous cell cancer (OC-SCC) while commensal *Streptococcus* spp. was decreased. These oral pathogens were the primary source for transcriptional stimulation of genes encoding HSP90A, TLR-1/2/4 ligands [82]. Kim et al. indicated that HSP90 could increase telomerase expression through promoter activation of human oral cancer cells. This expression can interact with the human telomerase reverse transcriptase (hTERT) promoter [83].

Dental caries is much dependent on dietary carbohydrates. The *S. mutans* can alter these carbohydrates to organic acids and reduce the pH [84].

Mucosal candidiasis, known as thrush [85], is a common disease in patients receiving high doses of chemotherapy or immunosuppressive agents and caused by *Candida albicans* (*C. albicans*) [49, 86]. A key point of *C. albicans* diseases is the yeast-to-hyphal transformation by phospholipases (PLs). This phospholipase is capable of destroying the junctions between epithelial cells and cell membranes [45]. The *C. albicans* penetrates the epithelial cells of mucosal membranes directly or by binding Als3 and Ssa1 of hypha to E-cadherin, epidermal growth factor receptors, and HER2 of cells [87]. Furthermore, aspartic proteinase 2 (Sap2), another *C. albicans*'s lytic enzyme, can protect the organism from immune system proteins such as salivary lactoferrin and immunoglobulins. Saps can activate inflammatory factor IL-1*β* in mucosal lesions [88]. Also, some external factors like antifungals can help to elevate dysbiosis. In immunocompromised patients, fluconazole can enhance *C. dubliniensis*. The *C. dubliniensis* is known as another germ in oral candidiasis and candidemia, which can increase, Saps expression [88–90].

Hepatocytes are the hepatitis B virus's primary host cells. The HBV infection is transmitted by blood or sexual activity [91]. Interestingly, the diversity of oral microbiota was decreased in HBV chronic liver disease (HBV-CLD) patients. In HBV-CLD, patients' *Fusobacterium*, *Treponema*, *Eubacterium*, *Parvimonas*, *Pseudomonas*, and *Filifactor* could be detected, which can induce an increased risk of periodontal disease. Indeed, the long-term course of HBV infection and gut-liver axis microbiome changes were the probable causes of oral microbiota alteration. This reduction led to dysbiosis in gut microbiota. In HBV-CLD patients, a high level of inflammation factors like IL-6 and IL-1*β* impaired the oral immunity system by increasing the abundance of *Fusobacterium* and *Treponema*, which attacked gut microbiota as opportunistic pathogens [39]. Immunodeficiency disorders or infections dysregulate the immune system and influence the balance of oral microbiota. In HIV patients, dominant oral organisms are correlated with CD4 T cell count [92].

## 5. Respiratory Microbiota and COVID-19

The primary transmission route of COVID-19 is respiratory droplets. It can also be transmitted through close contact [93, 94]. Human microbiota comprises viruses, phages, bacteria, and fungi [95]. It is believed that bacteria and fungi' coinfection play a notable role during COVID-19 [10]. For instance, comorbidity associated with severe COVID-19 is a chronic pulmonary disease (CPD) [96]. The airway microbiota composition is altered in CPD patients [97].

Zhou et al. reported the secondary infections and coinfections in COVID-19 patients [98]. Regularly, the human microbiota influences susceptibility to respiratory infections [99]. Microbiota compounds in the lung are altered in COVID-19 patients, and the changes may have an essential role in the COVID-19 immunity and severity [100]. Commensal bacteria can affect antiviral immunity activation, and probiotics can reduce the time duration and degree of respiratory viral infections [101]. Some Gram-positive bacterial microbiota like *Staphylococcus aureus* has been shown to prevent influenza virus infections [102]. In patients with influenza A and B admitted to the ICU, the percentage of invasive pulmonary aspergillosis (IPA) is higher than patients with severe pneumonia caused by other pathogens except for flu (19% versus 5%) [103]. Schauwvlieghe et al. reported that the 3-month mortality rate of influenza patients with and without the IPA is 51% and 28%, respectively [103]. Regarding the epidemiological data to decrease morbidity and mortality in COVID-19 patients, antifungal chemoprophylaxis and environmental measures could be proposed [104].

Oral health deterioration in COVID-19 patients due to external ventilation and subsequent complexities can be caused by hyposalivation, even affecting the lower respiratory tract, similar to aspiration pneumonia [105]. Impaired balance of oral microbiota arises from systemic treatments and changes in the intraoral environment and may lead to other problems [105]. The large populations in the oral and upper respiratory tract microbiotas are from the *Streptococcus* spp. [106]. *Streptococci* can metabolize carbohydrates in the fermentation process and yield acids, which has a role in dental caries progress by species like *S. mutans* [106]. Patients with COVID-19 have notable lung microbiota, especially with potential dysbiosis and divergence from healthy individuals [107]. *Streptococcus salivarius* (*S. salivarius*) is a predominant oral cavity microbiota [108]. Colonization of *S. salivarius* K12 strain reduces the occurrence of some viral upper respiratory tract infections; in SARS-CoV-2 patients, this field needs further investigation [107]. In a study published in 2003, the severe acute respiratory syndrome (SARS) patients had a secondary infection, including a high percentage of the *Pseudomonas aeruginosa*, *Staphylococcus* spp., *Stenotrophomonas maltophilia*, *Klebsiella terrigena*, and fungal [109]. Further research is needed to confirm how microbiota communication is changing post-COVID-19 infection, inter- and intrapersonally. The results of current studies related to microbiota in the COVID-19 patients are shown in Table 2.

## 6. Respiratory Microbiota Dysbiosis and COVID-19

**Figure 1:**
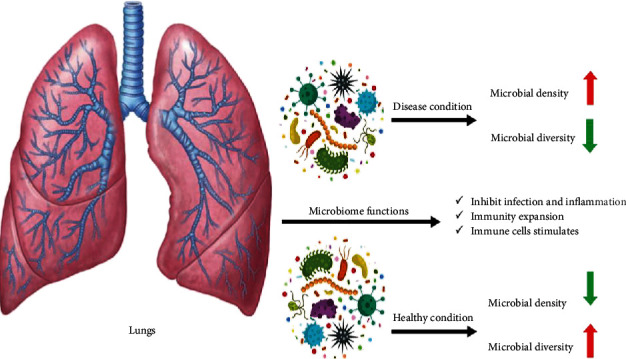
The lung microbiome in disease and health condition.

A neglected function of lung microbiota is the maintenance of immune tolerance, which leads to the prevention of inflammatory responses, helps lung homeostasis, and can also be supposed as lung health status [110]. The oral cavities are known as a notable reservoir of SARS-CoV-2 [111]. Since the oral microbiota interacts with SARS-CoV-2, efficient oral health care efforts are needed to reduce severe SARS-CoV-2 infections [112]. The microbiota in the human body, such as nasal channels, oral cavities, skin, gastrointestinal tract, and urogenital tract, are important in physiological process, immunity, and nourishment [113]. By recognizing crucial microbiota functions in human health and disease, it could be found that many complicated human disorders are correlated with microbiota [113, 114]. The schematic view of lung microbiota changes in disease and health conditions is conducted in Figure 1 [100]. With new insight into microbiota's role in human diseases and health, these findings can be implemented as a novel therapeutic target [115]. The healthy oral cavity's microbiota is distinct from bacterial inhabitants of other organs in human body. The human oral cavity comprises a distinct set of niches containing the tongue, tonsils, saliva, and teeth [116]. The same bacteria population organizes the oral microbiome in each healthy oral cavity niche [113, 116].

However, the microbiota is not uniform in different oral cavity circumstances. Bacterial diversity varies significantly between other sampling sites, including saliva, buccal mucosa, and back of the tongue supragingival plaque, and subgingival plaque [117].

Lung microbiota contributes to immunological homeostasis [110]. Viral infection may have considerable interplays with the commensal microbiota. Commensal microbiota can be altered by viral infections or even be reduced during infection [118].

Concerning COVID-19, a highly significant difference in the lung microbiota composition has been observed between patients with SARS-CoV-2 pneumonia and healthy population, implying a dysbiosis in patient's lung microbiota [119]. The Corynebacterium spp., Staphylococcus spp., Propionibacterium spp., and several *Malassezia* spp. have been recognized as the core nasal members microbiome already [120]. Chonmaitree et al. collected nasopharyngeal microbiota samples longitudinally during health and disease in infants [121]. The results suggested that bacterial otopathogen genera (*Haemophilus* spp., *Streptococcus* spp., and *Moraxella* spp.) were highly abundant in nasopharyngeal microbiota. These bacteria appear to correlate with upper respiratory tract infection (URI) symptoms during viral infection. Chonmaitree et al. mentioned the probiotic bacterium *Staphylococcus* spp. and *Bifidobacterium* spp. played a crucial role in inhibiting the otopathogens' harmful effects [121].

Respiratory microorganisms were widely characterized [42, 113, 122]. Balance in three factors, microbial immigration, microbial elimination, and relative reproduction rates, can determine lung microbiome characteristics [123]. The human respiratory tract harbors a homogenous microbiota that reduces biomass from the upper to the lower tract [42]. The nasopharynx core microbiome remains indistinct because it varies extensively from person to person in seasons [122]. One study reported that the upper respiratory tract's microbial balance is typically unique to each person, changing little over time [124]. However, the antimicrobial prophylaxis and treatment may induce dysbiosis in airway microbiota and increase the *Haemophilus parainfluenzae* and yeast colonization [125].

By increasing mucosal function and the ability to differentiate structure, stimulating in both the innate and adaptive immune systems, and giving “colonization resistance” against pathogen invasion, the human microbiota is regarded to benefit the host [126]. The commensal microbiota's importance was described in viral infection, with the commensal microbiota composition critically regulating host immune response following respiratory infections such as influenza A virus [127]. A wide range of respiratory tract infections is caused by viruses, including coronavirus, rhinovirus, respiratory syncytial virus, and influenza virus [128]. Infection by respiratory viruses has a pathological effect on the respiratory tract caused by the viral invasion or immunopathogenesis process and induced microbiome alterations and secondary infection [18, 129, 130]. Lei et al. reported that monitoring fungal infection in patients with SARS-CoV-2 should be considered due to the high positive rate of fungal antigenemia [131]. Also, Chen et al. reported fungal coinfections, including *C. albicans* and *C. glabrata*, between patients with COVID-19 [10]. Preliminary reports showed further investigations need to evaluate fungal coinfection among COVID-19 patients [10, 131]. In one study beginning in the outbreak and with the fast spread of the SARS-CoV, the first few cases were treated with a mixture of ribavirin and corticosteroids, with good results. Long-term treatment with high-dose steroids and the lack of an active antimicrobial agent can cause difficulties such as disseminated fungal infection in patients [132]. Corticosteroid therapy, which is usually sufficient to modulate immune reaction in severe inflammatory conditions, seems harmful in some of the COVID-19 cases [133, 134]. Fungal and bacterial infections are common complications of viral pneumonia in seriously ill patients [135]; a comprehensive investigation is needed in COVID-19 patients.

## 7. Respiratory Microbiota and COVID-19 Transmission

Yildiz et al., in an experience of influenza A virus infection on a mouse model, indicate qualitative dysbiosis and bacterial superinfection sensitivity in the lower respiratory tract microbiota compounds [136]. Observing overall shifts in the bacterial and fungal community of sinus diversity was shown to be attributed to a compound of personal, seasonal, and annual changes [120]. Oral opportunistic pathogens like *Capnocytophaga* and *Veillonella* were found in the bronchoalveolar lavage (BAL) sample of the COVID-19 patients [112]. The poor oral hygiene, cough, raised inhalation conditions, and ventilation cause a transmission route for oral microbiota to penetrate the lower respiratory tract and cause respiratory disorders [112].

During COVID-19, some pathological oral conditions could be aggregated, especially in the compromised immune system and prolonged therapeutic approach [105]. Appearing evidence submits that the nasopharyngeal microbiota's composition is correlated with susceptibility to acute respiratory infections and, importantly, the host immune response in children [137]. It has been shown that respiratory tract bacteria are not inactive during severe respiratory infections but rather have a complex interaction with the host immune response and infecting viruses [138, 139]. Ecosystem imbalance may cause overgrowth and invasion by bacterial pathogens and beginning respiratory or invasive diseases [140]. Respiratory bacteria and respiratory viruse colonization is frequently competitive interspecies interactions and can induce microbiota dysbiosis at the nasopharyngeal niche [140].

SARS-CoV-2 infection likely occurs in patients already colonized with bacteria. Besides, the very reasonable possibility exists that severe COVID-19 patients could be subsequently or coincidentally infected by bacteria and fungi [10]. In COVID-19, detecting bacterial or fungal infection based on the clinical and radiological form could be challenging. The microbiological techniques can help diagnose, mainly sputum culture [135]. The bacterial composition of the nasal microbiota varies between stages of life [141]. A cross-sectional study focused on this transition indicates that puberty has a significant impact on nasal microbiota composition. There are statistically significant differences in nostril microbiota compounds, in which *Actinobacteria* spp. and particularly *Corynebacterium* spp., *Propionibacterium* spp., and *Turicella* spp. are overrepresented in some conditions [142]. By affecting COVID-19 on most of the ciliated cells in the alveoli and disturbance on clearing the airways, progressive debris and fluid accumulation could be expected [143].

## 8. Respiratory Microbiota and COVID-19 Severity

The human upper respiratory tract is the leading entrance for aerosol-transmitted microorganisms, including SARS-CoV-2 [144]. The complex interactive oral microbiota has an expansive biofilm configuration. Besides the bacteria, *Candida* is a typical microbiota. Also, 100 recognized species of pathogenic fungi, including *Cryptococcus* spp., *Aspergillus* spp., and *Fusarium* spp., appear to reside in some individuals [145]. The microbiota of healthy lungs overlaps with that found in the mouth [146]. In bronchoalveolar lavage fluid samples from healthy adults, the well-known genera consist of *Streptococcus* spp., *Prevotella* spp., and *Veillonella* spp. are detected [146, 147]. Strain K12 of *Streptococcus salivarius* has been clinically demonstrated to play a role in creating a stable upper respiratory tract microbiota due to the ability to stimulate IFN-*γ* release and to activate natural killer cells (NK) without triggering aggressive inflammatory responses. Also, strain K12 is capable of protecting the host from pathogenic viral infections. The proposed antiviral capability of strain K12 has been attributed to the observed development of an adaptive immune response, as revealed by the detection of enhanced IFN-*γ* levels in human saliva [107]. More investigation needs to evaluate the impact of strain K12 on SARS-CoV-2 and COVID-19 severity.

The innate and adaptive immune systems are active against the SARS-CoV-2 infection. Lymphopenia, with an enormously decreased of B cells, CD4^+^ and CD8^+^ T cells, NK cells, and monocytes, is associated with the increased severity of COVID-19 [148, 149]. The regulatory T cells can affect microbiota and microbiota regulating the immune system and play an essential role in maintaining homeostasis [150, 151]. Gathering obtained evidence with different mediations such as antibiotic exposure, and microbiota transfer showed that the microbiota could enhance antiviral immunity, a new perspective for efficient treatments in COVID-19 patients [19]. The SARS-CoV-2 mutations could cause alterations in virus pathogenicity [152]. Hence, it is crucial to investigate the pattern and rate of mutations that happened [153].

Lung microbiota is associated with disease susceptibility and severity [154]. Shen et al. analyzed changes in the lung microbiota composition in SARS-CoV-2-infected patients and showed the microbial balance in these patients' BAL. Commensal and pathogenic bacteria dominate this communication, and this composition is also different from the healthy control group [119]. Few studies have been performed on the interaction between lower respiratory tract (LRT) microbiota and viral infections. Alterations in the microbiota in the LRT during viral infection were variable and might result from the reduced capability to remove pathogens in the upper respiratory tract [19]. Probiotics can develop immunity against influenza infection. The microbiota can probably work as a target for antiviral therapy [19]. It needs to be understood how microbiota could help assess clinical status and serve as a target for anti-SARS-CoV-2 therapies [19].

## 9. Conclusion

Microbiota communities play critical roles in immune system homeostasis. Therefore, any alteration in the healthy humans' microbiota can have detrimental impacts on health and may lead to an infection or the progression of the disease. It seems that the microbiota balance differs between the healthy group and COVID-19 patients. Dysbiosis in certain microbiota species' populations may alter the pathogenesis of COVID-19 in patients. Therefore, tracking these changes is useful as a prognostic approach during COVID-19 treatment. Further studies are needed to determine significant cellular changes resulting from SARS-CoV-2 and microbiota interactions.

## Figures and Tables

**Table 1:** The predominant human oral microbiota.

Sites	Microbiota	Ref
Lips	*Streptococcus* spp., *C. Albicans*	[30]
Hard palate	*Streptococcus* spp., *Uncl. Pasteurellaceae*, *Mogibacterium Veillonella*, *Catonella Prevotella*, *Uncl. Lactobacillales*, *Gemella*	[21]
Tongue	Front two-thirds of the tongue: *Streptococcus mutans*	[25, 31]
	Tongue dorsum: *Streptococcus salivarius*, *S. oralis*, *S. mitis*, *Actinomyces naeslundii*, *Haemophilus* spp., *Rothia mucilaginosa*	
Gingival sulcus	*Proteobacteria* (genus *Acinetobacter*, *Haemophilus*, *Moraxella*), *Firmicutes* (*Streptococcus*, *Granulicatella*, *Gemella*)	[32]
Buccal mucosa	*Firmicutes* (*Streptococcus sanguinis*, *S. oralis, S. mitis*)	[31, 155]
Palatine tonsils	*Streptococcus*, *Prevotella*, *Neisseria*, *Fusobacterium*, *Veillonella*	[156]
Saliva	*Firmicutes* (genus *Streptococcus* and *Veillonella*), *Bacteroidetes* (genus *Prevotella*), and *Betaproteobacteria* (genus *Neisseriaceae*)	[25, 31, 157]
Teeth (dental plaque)	Tooth crown: *Firmicutes* (genus *Streptococcus* and *Veillonella*)	[25, 31, 32]
	Supragingival plaque: *Firmicutes* and *Actinobacteria* (genus *Corynebacterium* and *Actinomyces*)	
	Subgingival plaque: *Obsidian Pool OP11*, *TM7*, *Deferribacteres*, *Spirochaetes*, *Fusobacteria*, *Actinobacteria*, *Firmicutes*, *Proteobacteria*, *Bacteroidetes*, *C. albicans*	

**Table 2:** The predominant microbiota in the COVID-19 patients reported from current studies.

Type	Outcome	Ref
*Acinetobacter*, *Chryseobacterium*, *Burkholderia*, *Brevundimonas*, *Sphingobium*	The critical impact of mucosal microbiota on the susceptibility to SARS-CoV2 infection and severity of COVID-19 patients	[158]
*Cutaneotrichosporon*, *Issatchenkia*, *Wallemia*, *Cladosporium*, *Alternaria*, *Dipodascus*, *Mortierella*, *Aspergillus*, *Naganishia, Diutina*, *and Candida*		
*Firmicutes* (42%), *Bacteroidetes* (25), *Proteobacteria* (18%), *Actinobacteria* (8%), and *Fusobacteria* (5%)	No statistically significant differences in nasopharyngeal microbiota of SARS-CoV-2 infection.	[144]
*Acinetobacter* (80.70%), *Chryseobacterium* (2.68%), *Burkholderia* (2.00%), *Brevundimonas* (1.18%), *Sphingobium* (0.93%), *Mycobacterium* (3.59%), and *Prevotella* (0.56%)	COVID-19 mortality is associated with complex mixed bacterial and fungal infections in the lungs, and microbiota monitoring is necessary in the lower respiratory tract for on-time personalized therapy.	[159]
*Cutaneotrichosporon* (*Cryptococcus*, 28.14%), followed by *Issatchenkia* (8.22%), *Wallemia* (4.77%), *Cladosporium* (4.67%), *Alternaria* (4.46%), *Dipodascus* (4.01%), *Mortierella* (3.22%), *Aspergillus* (2.72%), *Naganishia* (2.53%), *Diutina* (2.15%), and *Candida* (1.42%)		

## Data Availability

All data associated with this manuscript is inclusive in this paper.

## Additional Points

All tables and figure in this study are original.
